# Supplementary material for: Novel therapies for cancer-induced bone pain
Source: Neurobiol Pain. 2024 Sep 26;16:100167. doi: 10.1016/j.ynpai.2024.100167 (PMC11470602; doi:10.1016/j.ynpai.2024.100167)
Supplement: Supplementary Data 1 [file mmc1.docx]

# Supplementary figures


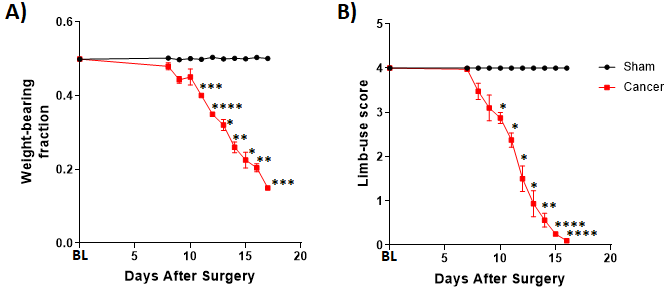


Supplementary figure 1: CIBP is associated with a reduction of the weight-borne on the affected limb (A) and its use score (B). N=5 in the sham group and 4 in the cancer group at the baseline. Error bars represent the SEM. The RMEL model indicates that the cancer group has significantly lower limb-use and weight-bearing scores than the sham group over time (p-value <0.0001 in both tests). All mice included in this study were males. The asterisks of significance shown in the figure represent the post hoc analysis results comparing the two groups at each time point.
